# Early Detection of Cerebral Embolism During Transcatheter Aortic Valve Replacement Facilitated by Rapid Awakening Through the Antagonism of Remimazolam With Flumazenil: A Case Report

**DOI:** 10.7759/cureus.77131

**Published:** 2025-01-08

**Authors:** Atsuhiro Kitaura, Yukari Yoshino, Hiroatsu Sakamoto, Shota Tsukimoto, Yasufumi Nakajima

**Affiliations:** 1 Anesthesiology, Kindai University Faculty of Medicine, Osaka, JPN; 2 Anesthesiology, Kindai University Faculty of Medicine, Osakasayama, JPN; 3 Anesthesiology, Kindai University Faculty of Medicine, Osaka-Sayama, JPN; 4 Dental Anesthesiology, Kanagawa Dental University, Yokosuka, JPN; 5 Anesthesiology and Center for Outcomes Research, University of Texas Health Science Center, Houston, USA

**Keywords:** cerebral embolism, cerebral infarction, flumazenil, monitored anesthesia care, quality of recovery, remimazolam, transcatheter aortic valve replacement

## Abstract

The combination of remimazolam and flumazenil facilitates the rapid acquisition of neurological findings immediately after awakening from anesthesia, potentially improving the likelihood of early detection of neurological complications during anesthesia. Cerebral infarction is a serious complication of transcatheter aortic valve replacement (TAVR), which can significantly impact a patient’s quality of life and activities of daily living. Although complete prevention of cerebral infarction remains challenging, early detection and prompt treatment can improve the neurological prognosis.

We present the case of an 83-year-old woman in whom a stroke was diagnosed immediately after TAVR. Prompt transcatheter revascularization was performed, leading to a favorable neurological outcome. The patient underwent transfemoral artery TAVR under sedation with remimazolam and remifentanil. A 26-mm Evolut Pro+ valve was placed in the planned position, and implantation was completed successfully. Following TAVR, the patient was awakened with flumazenil. Due to poor arousal, aphasia, and right-sided muscle weakness, she was referred to the neurology department for further evaluation. Approximately 10 minutes after the surgery was completed, a decision was made to perform a detailed evaluation of a potential stroke in the operating room. Imaging revealed an occlusion in the inferior division of the left middle cerebral artery. Successful recanalization was achieved through catheter-based thrombectomy 200 minutes after the procedure. The patient’s postoperative course was favorable, except for some aphasia. She was discharged and able to live independently, without difficulty, similar to her condition before surgery.

To detect cerebral infarction early, it is crucial to assess neurological status post-TAVR promptly. Rapid awakening from anesthesia and distinguishing between the effects of anesthesia and neurological deficits are essential. The combination of remimazolam and flumazenil can effectively and rapidly reverse the effects of anesthesia within about one minute after flumazenil administration, potentially facilitating the early detection of a stroke.

## Introduction

Cerebral infarction is a major complication of transcatheter aortic valve replacement (TAVR) [1-3]. Once a stroke occurs, mortality is significantly increased, and the possibility of returning to society is low [4]. The dispersal of emboli by the TAVR procedure is the most common cause of cerebral infarction during TAVR [5]. Currently, cerebral infarction during TAVR cannot be completely prevented, even with the selection of an appropriate approach and careful technique. At the same time, it is possible to make technical adjustments such as selecting the correct approach, careful device delivery, and minimizing wire manipulation. Therefore, catheter-based embolic retrieval is the main treatment for post-TAVR cerebral infarction; however, the indication for this procedure is limited [6]. Therefore, neurological findings should be confirmed as soon as possible after the TAVR procedure, and if abnormalities are found, the patient should be referred to a neurologist to determine the cause. When assessing postoperative neurological findings, the residual effects of anesthetics often make this difficult.

Remimazolam, a new ultrashort-acting benzodiazepine, may be advantageous for postoperative neurological diagnosis because an antagonist with clinical certainty exists and can allow for reliable and rapid arousal [7-9]. So far, the utility of remimazolam in TAVR has not been established. Since its release, we have been using remimazolam for sedation during TAVR. In our experience, we have reported that with the use of flumazenil, anesthesiologists can achieve awakening in most cases within about one minute after attempting to rouse the patient [10]. The combination of remimazolam and flumazenil often results in the patient becoming fully conscious immediately after awakening, with the ability to follow commands, enabling the assessment of neurological abnormalities in the operating room. Therefore, if a cerebral infarction is suspected, this approach may allow for a prompt transition to the next necessary steps. Herein, we report a case of cerebral infarction due to a cerebral embolus after TAVR performed under remimazolam-based sedation, in which a good prognosis was maintained by prompt response, and provide a review of some related literature.

## Case presentation

**Figure 1:**
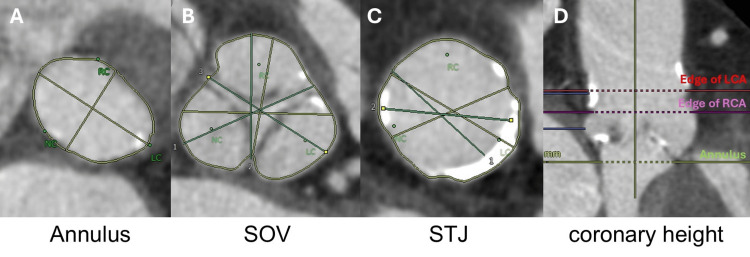
Preoperative CT of the patient’s aortic root LCA: left coronary artery; RCA: right coronary artery; SOV: sinus of Valsalva; STJ: sinotubular junction. A: Annulus level short-axis view. The annulus area was 319 mm^2^, and the valve circumference was 66 mm. B: SOV level short-axis view. The SOV sizes were 27, 28, and 30 mm. C: STJ level short-axis view. The STJ diameter was 23 x 24 mm and was highly calcified. D: Long axis view of aortic root. The LCA height was 19.7 mm, and the RCA height was 13.7 mm. The structure of the aortic valve base was adapted to the 26-mm Evolut pro + device.

An 83-year-old woman (height, 150 cm; weight, 57 kg) presented with exertional dyspnea and was referred to our hospital for TAVR due to symptomatic severe aortic stenosis. Her medical history included atrial fibrillation, depression, bilateral total knee arthroplasties, right total hip arthroplasty, and left middle cerebral artery (MCA) aneurysm clipping. She had no neurological abnormalities and was independent in daily life. Her current medications included sacubitril/valsartan 200 mg/day, bisoprolol 2.5 mg, apixaban 5 mg, lemborexant 5 mg, and escitalopram. Blood tests revealed mild renal dysfunction (estimated glomerular filtration rate, 56.5 mL/min/1.73 m²) but no other abnormalities. Preoperative echocardiography showed an aortic valve area of 0.93 cm², peak aortic jet velocity of 4 m/s, left ventricular ejection fraction of 74%, and absence of wall motion abnormalities and other valvular issues. Computed tomography (CT) revealed an annular area of 319 cm², a valve perimeter of 66 mm, and tricuspid valve leaflets with calcification. The sinus of Valsalva (SOV) ranged from 27 to 30 mm, and the sinotubular junction ranged from 23 to 24 mm, with moderate calcification (Figure 1).

**Figure 2:**
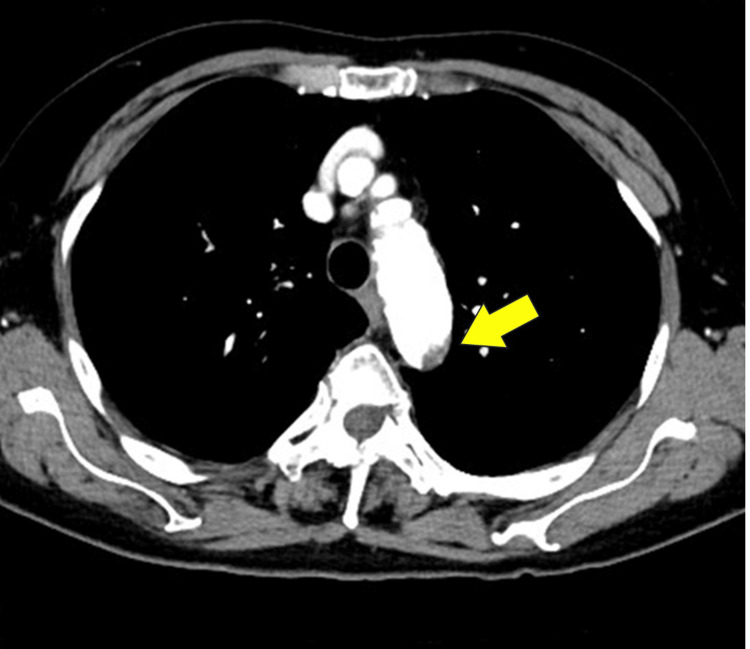
Preoperative enhanced CT of the patient’s aortic arch The arch aorta is slightly shaggy, and a plaque in the wall (arrow) is seen on the arch aorta’s greater fold side.

The access diameter was adequate, and the transfemoral approach was considered feasible. However, plaques were found in the aortic arch (Figure 2).

Given the narrow aortic annulus, an Evolute Pro+ 26-mm valve was selected.

On the day of surgery, only sacubitril/valsartan was discontinued, while the other oral medications were continued. Intraoperatively, after attaching basic monitors (blood pressure cuff, electrocardiogram, and transcutaneous arterial oxygen saturation monitor), a right radial arterial pressure line was inserted under local anesthesia with 1 mL of 1% lidocaine. Sedation was performed with remimazolam and remifentanil according to our facility's protocol. Our facility's protocol was selected as the best approach based on the results of using various anesthetics, including commonly used drugs such as propofol and dexmedetomidine for sedation during TAVR. The remimazolam-based sedation protocol used for this patient was chosen based on the result that it had less impact on hemodynamics and allowed for rapid awakening compared to protocols based on dexmedetomidine and propofol [10].

After the intravenous administration of 0.1 mg/kg remimazolam, the patient was asleep, and anesthetic maintenance was achieved with 1 mg/kg/h remimazolam and 0.03 µg/kg/h remifentanil. The depth of sedation was maintained within the range of 40-70 on the bispectral index. Before starting the procedure, 1000 mg of acetaminophen, 6.6 mg of dexamethasone, and 1 mg of granisetron were administered. Heparin (100 units/kg) was administered, and the activated clotting time was >250 s. Balloon predilatation was performed with an 18-mm Inoue balloon (Toray Co., Japan), and the device passage was smooth. The valve deployment was performed under ventricular pacing (VVI: 120); however, valve positioning required several adjustments and re-captures, completing the deployment after three attempts. Post-deployment aortic angiography showed moderate perivalvular leakage from the left coronary cusp side. A postdilation procedure was performed with 23-mm Z-med balloon catheter (Numed Canada Inc., Canada) under ventricular rapid pacing (VVI:180). On aortic angiography after post-dilation, the perivalvular leakage (PVL) improved to the mild range, and at this point, anesthesia was terminated. Protamine (1 mg/kg) was administered, and compression hemostasis was applied at the puncture site. The TAVR procedure took 71 min. The sedation time was 102 min.

**Figure 3:**
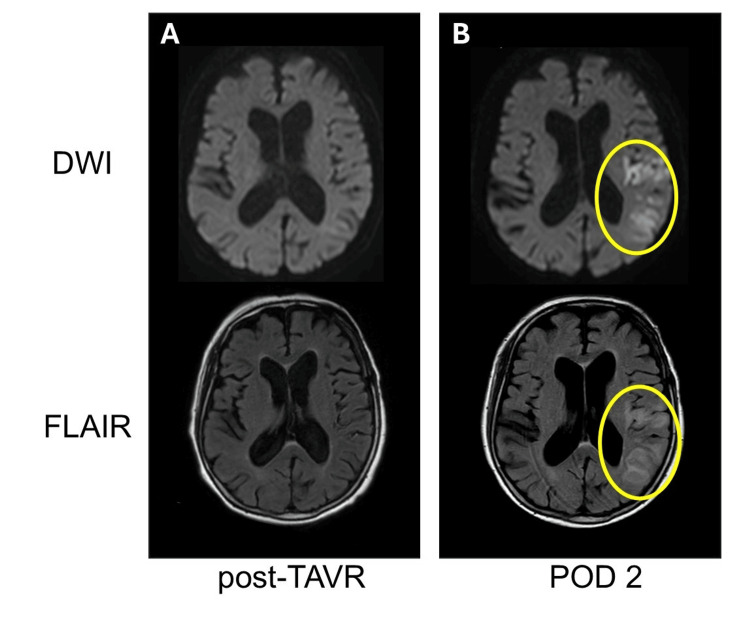
Magnetic resonance images after TAVR and on the second postoperative day DWI: diffusion-weighted image; FLAIR: T2-weighted fluid-attenuated inversion recovery image; POD: postoperative day; TAVR: transcatheter aortic valve replacement. A: The post-TAVR images showed no significant changes in both DWI and FLAIR. B: The POD2 images showed high-signal areas along the ischemic region both DWI and FLAIR.

Ten minutes after the surgery, flumazenil 0.5 mg was administered to reverse the anesthesia, and the patient opened her eyes within a minute. She responded “Hai” (“Yes” in English); however, other speech was absent, and her right forearm movement was impaired. After another 0.5 mg of flumazenil was administered, no change was observed, raising the suspicion of a cerebral infarction. Normally, the decision to perform a magnetic resonance imaging (MRI) is made by the neurologist during the consultation in the postoperative intensive care unit. However, as an exception, the decision was made to perform an MRI and consult with the neurologist 13 minutes after the surgery in the operating room. On neurological examination performed by the neurologist while waiting for the MRI, 25 minutes after the surgery, the patient had a score of 13 (E4V3M6) on the Glasgow coma scale, incomplete left conjugate deviation of the eyes, and right spatial neglect. She had manual muscle testing (MMT) scores of 0/5 in the right upper extremity and 1/5 in the right lower extremity, and a National Institutes of Health Stroke Scale score of 19 points. Her MRI was performed 50 min after surgery. Diffusion-weighted imaging (DWI) and T2-weighted fluid-attenuated inversion recovery imaging (FLAIR) showed no significant changes (Figure 3).

**Figure 4:**
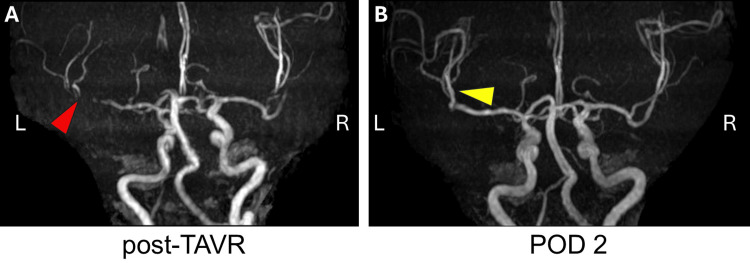
Magnetic resonance angiography after TAVR and on the second postoperative day POD: postoperative day; TAVR: transcatheter aortic valve replacement. A: The post-TAVR image. The inferior decision of the left middle cerebral artery disappeared (red arrow). B: The POD2 image. The occluded artery reappeared (yellow arrow).

However, magnetic resonance angiography revealed occlusion of the inlet of the left middle cerebral artery (MCA) inferior division (Figure 4). As a result, by performing imaging studies more promptly than usual, cerebral vascular occlusion was diagnosed at an earlier stage, before changes appeared on the MRI.

**Figure 5:**
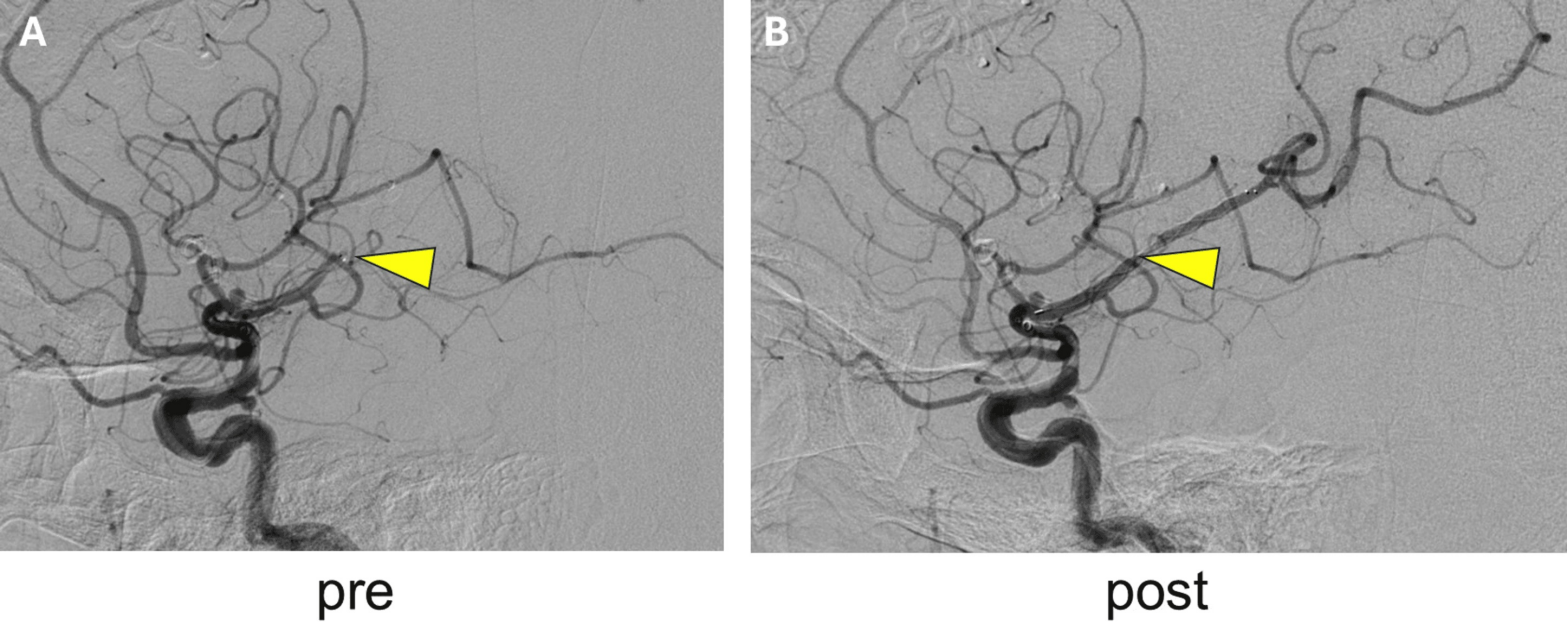
Left internal carotid arteriography before and after the catheter intervention A: Pre-interventional imaging. The left middle cerebral artery inferior division is occluded at the M2 level (arrow). B: Post-interventional imaging. The occluded vessel has been opened (arrow), and the peripheral side is contrasted.

The neurosurgery team was consulted, and an emergency thrombectomy was performed. One hundred minutes after the procedure, catheter intervention began in the catheterization room. In the left internal carotid artery (IC) angiography, a clip was observed in the left MCA, an aneurysm on the posterior wall of the left IC C2 segment, and occlusion of the MCA inferior division. It was believed that the MCA clip was not particularly involved in this event. While being cautious of the IC aneurysm, the distal side of the occlusion site was secured with a wire, and thrombectomy was performed twice using a stent and aspiration. Finally, the left MCA inferior division was completely recanalized (thrombolysis in cerebral infarction scale 3) 3 h and 20 min after the TAVR (Figure 5). Following recanalization, the right upper and lower limb strength improved to MMT 4/5, and the patient was able to respond to her name.

**Figure 6:**
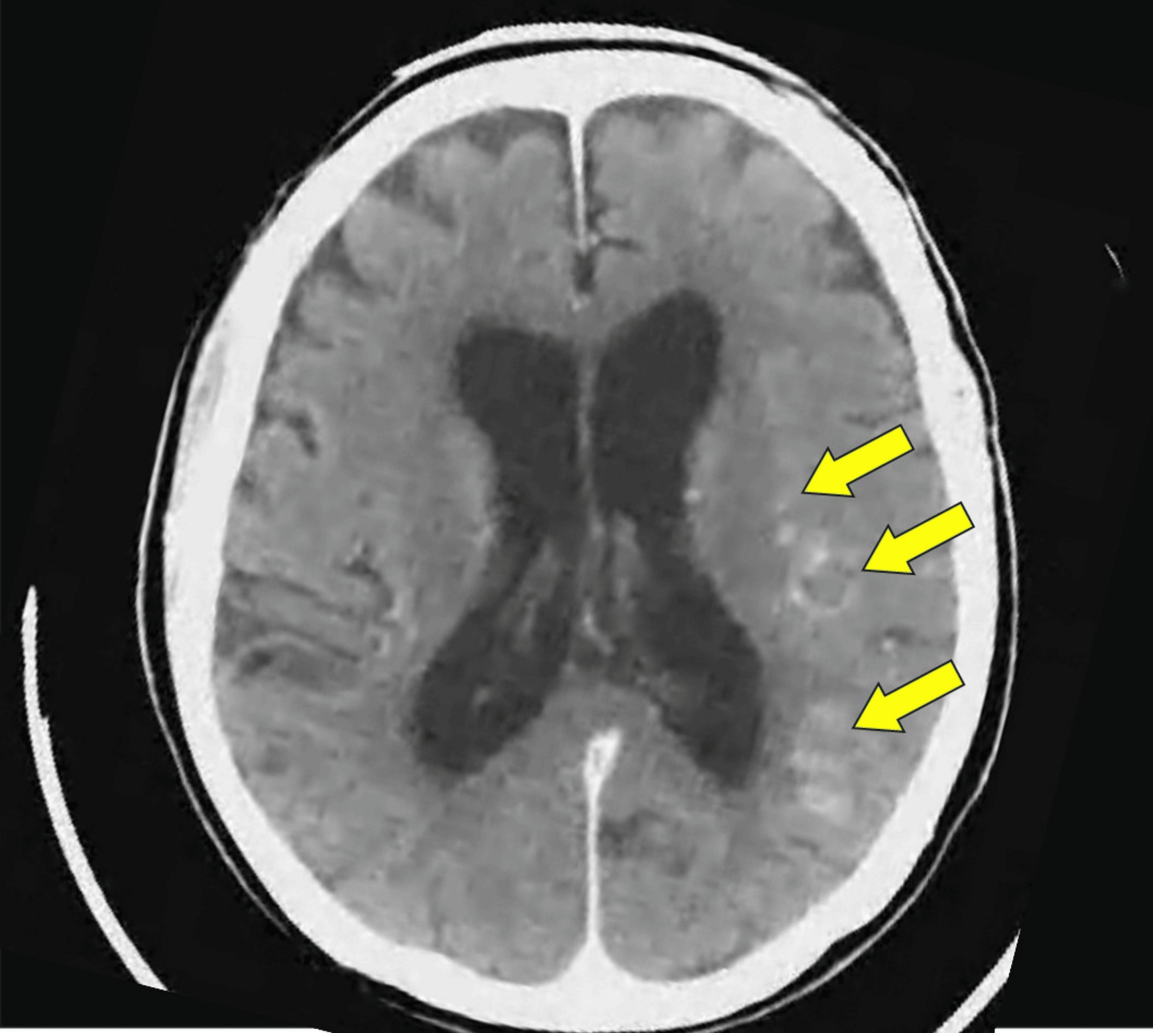
Computed tomography after reperfusion on the day of surgery A slight cerebral hemorrhage or contrast accumulation is observed in the ischemic region (arrows).

The pathological diagnosis of the tissue retrieved with the catheter was a mixed thrombus with cholesterol clefts. CT after reperfusion on the TAVR day revealed minimal hemorrhage or contrast accumulation in the ischemic region (Figure 6).

The next day, the MRI showed enlargement of the infarction area and reappearance of occluded vessels (Figures 3, 4); however, her motor function improved to pre-surgery levels immediately after cerebral reperfusion therapy. There was slight sensory aphasia to the extent that the patient was aware of some speaking difficulty; however, communication was good. During the hospital stay, speech rehabilitation was provided by a specialized physical therapist, and both language and physical functions were evaluated over time. After 14 days of rehabilitation, the patient was discharged. One month after TAVR, it was confirmed during her outpatient visit that she was able to live independently and communicate smoothly.

## Discussion

In this case, a stroke occurred during the TAVR procedure; however, because neurological abnormalities were detected immediately after surgery, a thrombectomy was performed promptly, leading to a favorable neurological outcome for the patient. Anesthesia with the combination of remimazolam and flumazenil, which allowed for rapid loss of anesthetic effect, contributed to the detection of neurological abnormalities in the immediate postoperative period. The results showed that the patient was able to move on to close examination and treatment of cerebral infarction within ten minutes after her TAVR procedure, which was quicker than the usual process.

Stroke is one of the most feared complications of aortic valve replacement. Although the outcomes of transcatheter aortic valve implantation (TAVI) have significantly improved over time, the frequency of symptomatic stroke within 30 days after surgery remains 0.6%-2.5% [1-3]. The mortality rate for patients who experience a stroke after TAVR is three to nine times higher than those who do not, and even survivors often require long-term care. Furthermore, approximately 80% of patients who had a post-TAVR stroke face difficulties returning to society. Consequently, a study suggested that patients fear stroke more than death [4]. In this case, the vessel supplying a relatively large area of the dominant hemisphere was occluded. Paralysis of the right side of the patient’s body and aphasia have seemed to be caused by this. If prompt recanalization had not been achieved, the patient would have likely developed an extensive cerebral infarction in the MCA region of the dominant hemisphere, potentially leading to significant impairments in daily living postoperatively. In fact, upon awakening from anesthesia, the present case had motor impairment of the right side of her body and aphasia, and it was estimated that without improvement, independent daily living would have been almost impossible. Multiple steps during TAVR can lead to cerebral embolism. Although not all patients present symptoms, embolism occurs in nearly every patient undergoing TAVR [5,10]. The embolus in this case was also a cholesterol embolus, and it was considered caused by the dispersal of the known aortic plaque during the surgical procedure. DWI after TAVR showed new lesions in 94% of cases, with an average of 10.4 ± 15.3 lesions per patient [10]. The necessity of using filter devices is still being debated but is not yet widespread [11]. Thus, cerebral embolism remains an unavoidable and serious complication of TAVR.

There is a time limit for treating symptomatic cerebral embolism. Thrombectomy is recommended within 6 h of onset [6], with better outcomes when treatment is initiated sooner [12]. In the United States, target times for initiating treatment are also set [13]. Indications for revascularization therapy are generally limited to periods when a DWI-FLAIR mismatch is present. In the previous case, by detecting and treating the cerebral infarction at an extremely early stage, where minimal changes were even observed on DWI, we were able to salvage a certain portion of the territory supplied by the occluded vessel. Consequently, the patient’s neurological deficits were kept to a minimum. Therefore, promptly identifying treatable strokes after TAVR is crucial. If the neurological abnormality was unclear, the patient could be followed up for a while for suspected residual effects of the anesthetic. Especially for procedures such as TAVR, where multiple surgeries are often scheduled on the same day by the same surgical team, there is a risk that the decision may be postponed until after the next surgery, thus missing the indication for reperfusion therapy. To facilitate this, rapid recovery from anesthesia and neurological assessment post-surgery is highly desirable.

TAVR is commonly performed under sedation; however, general anesthesia may also be used [14]. Although short-acting sedatives have become more popular, there are no antagonists for traditional intravenous anesthetics such as dexmedetomidine or propofol. In addition, volatile anesthetics, though rapidly eliminated from the body, can persist for half a day, potentially causing postoperative agitation [15]. This makes distinguishing between anesthesia effects and neurological disorders difficult in delayed awakening cases. Remimazolam, a very short-acting benzodiazepine [7,8], has minimal effects on circulation [7,8] and can be rapidly reversed using flumazenil [9]. A study has shown that patients sedated with remimazolam awaken significantly faster than those sedated with dexmedetomidine or propofol [16]. In fact, nearly all patients in studies involving remimazolam regained the ability to follow commands within a minute of receiving flumazenil [16]. In other words, failure of rapid arousal or the presence of neurologic abnormalities after antagonism by flumazenil is sufficient evidence to strongly suspect an organic abnormality other than anesthesia. Given that the rapid identification of treatable strokes is crucial, in light of this case, remimazolam appears to be a useful anesthetic for TAVR procedures. However, this report is a single case report. Therefore, it is not feasible to even mention drug comparisons based on this report alone. Future comparative studies are expected to determine whether the combination of remimazolam and flumazenil is useful.

## Conclusions

We experienced a case where the combination of remimazolam and flumazenil effectively and rapidly reversed the anesthetic effects, which helped in the early recognition of stroke and contributed to a favorable neurological outcome. While further research is needed to draw definitive conclusions, remimazolam may become an attractive option as a sedative for TAVR patients at high risk for cerebral infarction.
